# Dual-acting antibacterial porous chitosan film embedded with a photosensitizer

**DOI:** 10.1080/14686996.2020.1795431

**Published:** 2020-08-03

**Authors:** Zi-Jun Liu, Shu-Ching Lin, Pei-Yuan Lee, Ying-Ting Lin, Zi-Lun Lai, Cheng-Chung Chang, Gou-Jen Wang

**Affiliations:** a Graduate Institute of Biomedical Engineering, National Chung-Hsing University, Taichung, Taiwan; b Department of Orthopedics, Show Chwan Memorial Hospital, Changhua, Taiwan; c Program in Tissue Engineering and Regenerative Medicine, National Chung-Hsing University, Taichung, Taiwan; d Department of Mechanical Engineering, National Chung-Hsing University, Taichung, Taiwan

**Keywords:** Dual-acting antibacterial film, porous chitosan/small molecular compound, bactericidal and bacteriostatic effects, 102 Porous / Nanoporous / Nanostructured materials; 211 Scaffold / Tissue engineering/Drug delivery

## Abstract

This study proposes to develop a dual-acting antibacterial film of porous chitosan (Cs) embedded with small molecular compound, which possesses photosensitive characteristics with bactericidal efficacy, to promote the accelerated recovery of infectious wounds. The Cs/small molecular compound (Cs-cpd.2) dressing was prepared using the freeze-drying method. Characterization of the synthesized Cs-cpd.2 indicated that it has high porosity and moisture absorption effect, hence enhancing the absorption of wound exudate. Experimental results showed that Cs-cpd.2 dressing has good bactericidal and bacteriostatic effects on *Staphylococcus aureus* under visible-light irradiation and has antibacterial effect in the dark. It was also found that the small molecular compound does not have cytotoxicity at a dose of 0–5 μM. Furthermore, Cs-cpd.2 that contained small molecular compound with a concentration of 0.3–1 μM has positive effect on both the cell viability rate and cell proliferation rate of human fibroblast CG1639. Cs-cpd.2 can significantly promote cell proliferation when the small molecular compound and the basic fibroblast growth factor bFGF were added together. Therefore, the proposed Cs-cpd.2 dressing is feasible for photodynamic therapy (PDT) and clinical wound dressing applications.

## GRAPHICAL ABSTRACT

**Figure:**
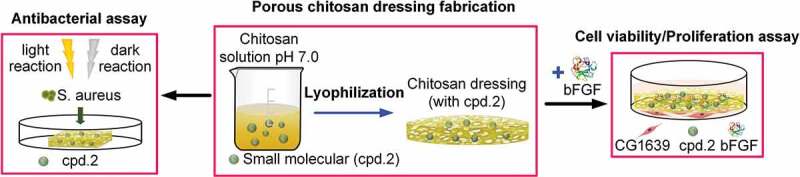

## 1. Introduction

Wound care is an important medical issue, and wound infection is often one of the main reasons that results in difficult recovery. Carelessness can cause cellulitis and sepsis and endanger patients’ lives. To prevent the infection of wound, antibiotics have been the most commonly used treatment. The excessive use of antibiotics has also led to the increasing resistance of bacteria to antibiotics. Common and important bacterial infections have developed resistance to first-line antibiotics, for example, vancomycin-resistant enterococci (vancomycin resistant; VRE). Enterococci (VRE) have become one of the important pathogenic bacteria in hospital infection groups in Europe and the United States.

To reduce the impact of antibiotic use, scientists have successively developed new antibacterial ingredients, methods, and dressings that effectively promote wound healing, allowing wounds to have better healing effects through the treatment with external dressings. Antibacterial dressings have been one of the hot research topics in the field of wound treatment. Thakur et al. [1] synthesized ZnO nanoparticle-loaded sodium alginate gum acacia (SAGA-ZnONPs) hydrogels and demonstrated that the proposed SAGA-ZnONP hydrogels significantly reduced the toxicity of ZnONPs and preserved the healing effect at a low concentration of ZnONPs. Zhao et al. [2] proposed an antibacterial, anti-oxidant, and electroactive dressing based on quaternized chitosan (Cs)-g-polyaniline and benzaldehyde group-functionalized poly(ethylene glycol)-co-poly(glycerol sebacate). Experimental results showed that the proposed hydrogel dressing could prolong the lifespan of dressing and promote the in vivo wound healing process. Zhang et al. [3] developed a three-dimensional (3D) layered nanofiber sponge to enhance the interfacial interaction between the sponge and blood cells to accelerate hemostasis. The fabricated 3D sponge could promote the regeneration of functional dermis and the restoration of differentiated adipocytes during the early repair phase. Wu et al. [4] prepared a nontoxicity and antimicrobial hydrogel P(M-Arg/NIPAAm) and demonstrated that the proposed hydrogel could effectively accelerate the full-thickness wound healing process.

Photodynamic therapy (PDT) is currently one of the most promising methods for treating drug-resistant bacterial infections. PDT uses the photosensitizing effect of photosensitizers (PS) to produce toxic effects on bacteria. The photosensitizing effect is usually induced by visible light with a wavelength of 400–700 nm. When the irradiated ground-state PS is located on the bacteria or bacterial surface, it absorbs light and is excited to a singlet state (^1^PS). The excited state electrons are then converted into a lower energy triplet state (^3^PS) through an energy gap conversion, which easily collides with other molecules in the environment to produce reactive oxygen species (ROS) to lyse bacteria [5]. The path of PDT to generate free radicals is divided into two types: type I is the collision of ^3^PS with surrounding ground-state molecules that causes electron transfer. Superoxide anions (•O_2_^−^) are produced during the reaction of ground-state molecules with oxygen molecules. In the biological environment, •O_2_^−^ easily undergoes chain reaction to produce ROS, such as hydroxyl radical (•OH) and carbonate anion radical (•CO_3_^−^). Type II is a collision between ^3^PS and ground-state oxygen molecules, in which high-energy singlet oxygen (^1^O_2_) is formed by energy transfer from ^3^PS to O_2_ [6]. If PDT is conducted in an oxygen-rich environment, type II reactions are likely to occur. Given that most photo-sensitive substances need to be in an aerobic environment to produce PDT, most PDTs are mainly type II.

Given that PDT uses active oxygen free radicals and ^1^O_2_ to hunt and kill cells, it differs from traditional antibiotic sterilization principles [7,8]. Photodynamic sterilization-related studies have been reported, and photodynamic sterilization has been used to combat drug-resistant bacteria. The use of PDT to kill bacteria is less likely to cause a drug-resistant response. Wang et al. [9] developed a linear tetrapyrrole metal complex, which possessed a thiol functionality to facilitate conjugation to near infrared-absorbing gold nanoshells, as a PS for PDT against triple-negative breast cancer cells. Nie et al. [10] used electrospinning technology to fabricate nanofibers that combined polyacrylonitrile and biocompatible carbon quantum dots for antibacterial photodynamic inactivation. Broad antibacterial efficacy of the proposed nanofibers was confirmed by experiments. These characteristics decrease the invasiveness of PDT and enable efficient treatments of drug-resistant bacteria in a patient-friendly manner.

Chitosan is biopolymer with a low toxicity and high biocompatibility, biodegradability and moisture retention when the degree of deacetylation of chitin reaches about 50%. Since the structure of chitosan contains reactive groups such as amine (-NH_2_) and hydroxyl groups (-OH), chitosan can be easily modified and further used as a biomedical material. The positive charge of chitosan can interact with the surface molecules of microorganisms to produce an antibacterial effect. Because chitosan can be shaped as films, beads, fibers, and gels, it can be used in wound dressings, controlled release of drugs, tissue engineering, and food antibacterial agents [11].

In this study, we propose a dual-acting antibacterial film of porous chitosan (Cs) embedded with small molecular compound. The antibacterial film was fabricated using the freeze-drying method. Effective bacteriostatic and bactericidal activity of the fabricated antibacterial film on *Staphylococcus aureus* (SA) under visible-light irradiation and in the dark was investigated. Cytotoxicity assay of the proposed antibacterial film on human fibroblast CG1639 was performed.

## 2. Materials and methods

**Scheme 1.**
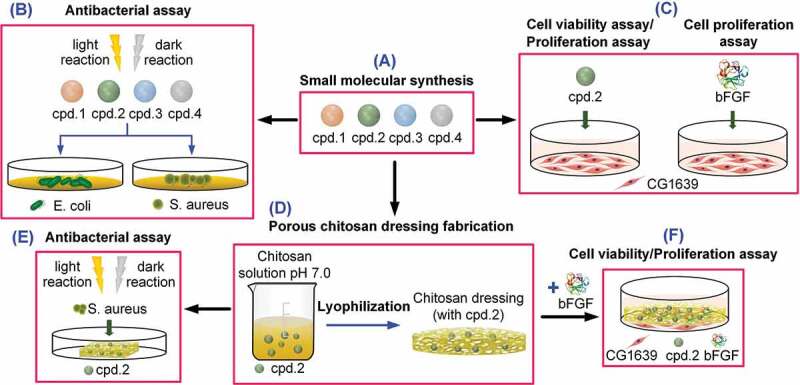
Schematic of the experimental process. (A) Small molecular compound synthesis; (B) antibacterial assay of small molecular compounds; (C) cell viability/proliferation assay of small molecular compounds and cell proliferation assay of bFGF; (D) porous Cs dressing fabrication; (E) antibacterial assay of porous Cs dressing; (F) cell viability/proliferation assay of porous Cs dressing.

Scheme 1 Outlines our experiment.

### 2.1 Antibacterial assay of small molecular compounds (Schemes 1(A) and 1(B))

#### 2.1.1. Materials

SA (BCRC 10,451) and *E. coli* (BCRC 11,634) were used in the antibacterial assay; four types of small molecular compounds (cpd.1, cpd.2, cpd.3, and cpd.4), which were reported in our previous works [12–14] and briefly described in the supplementary materials, were used as the photodynamic agents; nutrient agar (Merck kGaA, 1.05450.0500) and nutrient broth (Merck kGaA, 1.05443.0500) were prepared for bacterial culture.

*E. coli* and SA were removed from the −80°C refrigerator. After thawing the samples, a bacterial inoculation ring was used to pick up a small amount of bacterial solution and coat a slanted petri dish. The petri dish was placed in a 36°C incubator until the next day. The sample was stored at 4°C for further use.

#### 2.1.2. Antibacterial assay

In this study, the JIS-Z-1018 film material standard antibacterial method was slightly modified and used to examine the antibacterial effect of four small molecular compounds under and without visible-light irradiation. The experimental steps are as follows:

The bacteria stored in the refrigerator were removed and passaged twice every 24 h. The inoculation loop was used to scrape the bacteria that had passed through and spread them in 500-fold diluted lysogeny broth (LB). The absorbance was measured with a photometer. The bacterial solution was diluted 1000-fold to a concentration of 1 × 10^6^ colony-forming unit (CFU)/mL. Four kinds of small molecular compounds were prepared at 2, 1, and 0.5 µM concentrations in a dark environment by using a bacterial solution. A total of 400 µL small molecular compound mixed bacterial solution were obtained, injected into 48-well plates, and divided into light and dark reaction groups. The illumination group and dark reaction group, which was coated with aluminum foil, were placed under a light source (LI60, 420 nm, and 25 µW) for 50 min and in a 25°C incubator for 24 h. LB was used to serially dilute the bacterial solution in the 48-well plate, followed by the addition of 1 mL dilution to the culture bottle and mixed with the heated liquefied LA. After the LA was solidified, the samples were placed in a 36°C incubator, and the number of bacteria was calculated after 24 h using Equation (1) to estimate the antibacterial effect of small molecules. When log ≥ 2, the small molecules at this concentration, that is, the effective bacterial concentration, have a bacteriostatic effect greater than or equal to 99%.

(1) $$\log\frac{Control\;group\;colony\;number}{Experimental\;group\;colony\;number}$$

### 2.2 Preparation and characterization of porous Cs antibacterial dressing (*Scheme 1* *(D))*

#### 2.2.1. Materials and instruments

Experimental grade Cs (low molecular weight, CAS no. 9012–76-4, Sigma-Aldrich, USA), sodium hydroxide (Union Chemical Work LTD, Taiwan), and acetic acid (Choneye Pure Chemicals, Taiwan) were purchased. A freeze dryer (FDU-1200, EYELA, Japan) was used for freeze-drying fabrication of porous Cs antibacterial dressing.

#### 2.2.2. Preparation of porous Cs antibacterial dressing

Cs powder was added to a 1% acetic acid solution to prepare a Cs solution with a concentration of 1%. After the Cs powder was uniformly dissolved, 2% sodium hydroxide was added dropwise to the Cs solution until the pH was 7.0 ± 0.1. After the addition of cpd.2 (0 and 5 μM) and stirring with a magnetic stirrer for 30 min, 3 mL homogeneous mixed solution was placed in a Teflon mold (width × length × height, 2.2 × 2.2 × 2 cm), stored at −20°C for 24 h, and then retrieved. The solution was freeze-dried at −52°C and 15 Pa for 24 h. The prepared porous Cs antibacterial dressing was then stored in a moisture-proof box for subsequent use.

#### 2.2.3. Characterization of porous Cs antibacterial dressing

(a) Pore size measurement

We used field-emission scanning electron microscopy (SEM, JSM-6700 F, Jeol, Japan) to observe the surface morphology of the dressings. A total of 30 pores from the SEM image were selected. Image J software was used to estimate the pore size of the porous Cs antibacterial dressing.

(b) Porosity estimation

Equation (2) was used to estimate the porosity of the prepared antibacterial dressing:

(2) $$P=\frac{m_{2}-m_{1}}{\rho V}$$

where *m*_1_ denotes the dry weight of the dressing before soaking in alcohol, *m*_2_ is the wet weight of the dressing after soaking in alcohol, *ρ* is 95% alcohol density, and *V* is the volume of the dressing.

(c) Water absorption estimation

The dressing was soaked in 10 mL deionized water for 24 h. Equation (3) was used to estimate the water absorption of the prepared antibacterial dressing:

(3) $$W=\frac{w_{2}-w_{1}}{w_{1}}\times 100\%$$

where *w*_1_ denotes the dry weight of the dressing before soaking in deionized water, and *w*_2_ is the wet weight of the dressing after soaking in deionized water.

### 2.3 Cell viability assay (*Scheme 1* *(C))*

#### 2.3.1. Cell culture

Human fibroblast CG1639, provided by the Bioresource Collection and Research Center, Taiwan, were cultured in a T-75 flask (Corning 430,720) with a high-glucose Dulbecco’s Modified Eagle Medium (Gibco, USA) containing 15% fetal bovine serum (Gibco, USA) and 1% antibiotic-antimycotic (Gibco, USA) in an incubator at 37°C and 5% CO_2_. The medium was renewed every 2–3 days.

#### 2.3.2. Toxicity assay of cpd.2 on CG1639

CG1639 at 5 × 10^3^ cells/well was cultured in a 96-well plate for 16 h until the cells were completely attached to the bottom of the well plate. The culture solution was used to configure cpd.2 at different concentrations (0.3, 0.5, 1, 2, 5, 8, and 10 µM) and then added to a 96-well plate with adherent cells. After culturing for 24 h, cytotoxicity assay was performed.

#### 2.3.3. Cell proliferation assay of bFGF on CG1639

The culture medium was used to configure 1 mg/mL bFGF into different concentrations (0.5 1, 2, 4, 8, 16, 32, 64, and 128 ng/mL). CG1639 was then prepared AS a suspension, and cells with a concentration of 2 × 10^3^ cells/well were injected with bFGF at different concentrations and cultured in a 96-well plate. The culture solution containing bFGF was replaced every 2 days. Cell proliferation was estimated after 3 and 5 days of culture, respectively.

#### 2.3.4. Toxicity assay of porous Cs dressing to CG1639

(a) Dressing pretreatment

Pure Cs and Cs/small molecular compound (Cs-cpd.2) antibacterial dressings were prepared by using a 24-well plate as a mold, washed, and sterilized with UV light, 75% alcohol and PBS, placed in a 24-well plate, infused with culture medium, and stored in an incubator for later use. Before the experiment, the sterilized Cs and Cs-cpd.2 dressings were injected with 2 ng bFGF and then placed at room temperature for 2 h to stabilize.

(b) Cell seeding

The CG1639 was cultured in 24-well plate at the number of 2 × 10^4^ in each well. Cs, Cs-bFGF), and Cs-cpd.2-bFGF dressings were respectively placed in the cell-attached well plate with cell culture inserts, and the cell survival rate was measured after 24 h in the incubator.

#### 2.3.5. Proliferation assay of porous Cs antibacterial dressings on CG1639

(a) Dressing pretreatment

The pretreatment process was similar to that of the toxicity assay.

(b) Cell culture

The cell culture process was similar to that of the toxicity assay, except that the cell concentration was 8 × 10^3^ cells/well, and the culture time was 3 days.

#### 2.3.6. Dimethylthiazol-diphenyltetrazolium bromide (MTT) assay

The MTT powder was prepared as a 12 mM aqueous solution and stored at 4°C. After the removal of cells to be measured from the incubator, the culture solution and dressings were removed and washed with PBS. MTT and culture medium were added to the cells at a ratio of 1:10 in a dark environment and cultured at 37°C for 4 h. Then, the culture medium was removed, and dimethyl sulfoxide was added to dissolve formazan and measured the optical density (OD) value with an enzyme immunoassay analyzer (TECAN Sunrise ELISA Reader, Sunrise ™, Switzerland) at a wavelength of 550 nm. The OD value was calculated, and the cell survival rate was estimated by Equation (4):

(4) $$\%\;\;\;cell\;survival=\frac{OD_{t}-OD_{b}}{OD_{c}-OD_{b}}\times 100\%$$

where *OD_t_* is the value of the test group, *OD_b_* is the value of the blank, and *OD_c_* is the value of the control group.

### 2.4 Antibacterial assay of the porous Cs dressing (*Schemes 1(E)* *and 1(F))*

After the porous Cs dressing was successfully prepared, we used the JIS-L-1902 standard quantitative antibacterial test method to examine the antibacterial effect of Cs-cpd.2 and Cs dressings (control group) on SA and calculated the proliferation (F), bactericidal activity (L), and bacteriostatic activity (S) values. The experimental process is described as follows.

1. SA stored at 4°C was removed and subcultured once in 24 h. The process was repeated twice. The inoculation ring was used to scrape the completely cultured SA, which was spread in LB diluted 500 times. The absorbance was measured until 0.11 ± 0.01 with a photometer. The SA solution was diluted 200 times to 5 × 10^5^ CFU/mL. Cs and Cs-cpd.2 antibacterial dressings were placed in different culture dishes. Then, 200 µL of SA solution was dripped onto each piece of material.
2. The samples were divided into the control, light, and dark reaction groups. The control group was inoculated with Cs and Cs-cpd.2 antibacterial dressings for 0 h and 10 mL LB was injected immediately. Then, SA was washed out with vigorous shaking using a test tube shaker (Vortex Genie II), followed by serial dilution that was performed as follows. A total of 1 mL serially diluted SA solution was added to a petri dish and mixed with the heated liquefied LA. After the LA was solidified, the petri dish was placed in a 36°C incubator. The colonies were counted after 24 h.
3. The light group and the dark reaction group, which was coated with aluminum foil paper, were placed under a fluorescent light source for 50 min and then cultured at 36°C for 24 h. Next, SA was washed out, serially diluted, added to LA, and cultured at 36°C. After 24 h, the colonies were counted using Equation (5):

(5) $$\begin{array}{rl} & Proliferation\;value\;\left(F\right)=log\left(M_{b}/M_{a}\right)\\ & Bactericidal\;activity\;value\;\left(L\right)=log\left(M_{a}/M_{c}\right)\\ & Bacteriostatic\;activity\;value\;\left(S\right)=log\left(M_{b}/M_{a}\right)\\ & \;\;\;\;\;\;\;\;\;\;\;\;\;-log\left(M_{c}/M_{o}\right) \end{array}$$

where M_a_ is the number of bacteria in the Cs antibacterial dressing at 0 h, M_o_ is the number of bacteria in the Cs-cpd. 2 antibacterial dressing at 0 h, M_b_ is the number of bacteria in the Cs antibacterial dressing after 24 h culture, and M_c_ is the number of bacteria in the Cs-cpd. 2 antibacterial dressing after 24 h culture.

## 3. Results and Discussion

### 3.1 Antibacterial effect of small molecular compounds

The experiments were designed in accordance with the JIS-Z-1018 antibacterial testing specifications. In the experiments, Gram-negative and Gram-positive standard bacteria (*E. coli* (BCRC 11,634) and SA (BCRC 10,451), respectively), which are commonly observed in nosocomial infections, were used as test bacteria. The antibacterial effects of different concentrations of cpd.1, 2, 3, and 4 on *E. coli* and SA in the absence of light and under irradiation with visible light for 50 min were investigated. According to the JIS-Z-1018 antibacterial test specification, the sample has effective antibacterial properties when the antibacterial activity value is greater than or equal to Log 2 (that is, the antibacterial effect reaches 99%).

**Figure 1.**
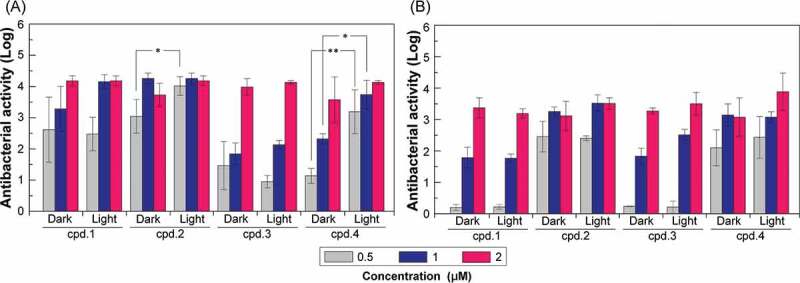
Antibacterial effect of small molecular compounds at different concentrations of SA (A) and *E. coli* (B) in the absence of light and under irradiation with visible light.

Figure 1(A) shows the antibacterial effects of cpd.1, 2, 3, and 4 at different concentrations of SA in the absence of light and under irradiation with visible light for 50 min. The results show that cpd.1 could effectively inhibit SA when the concentration was greater or equal to 0.5 μM when illumination has better antibacterial property. cpd.2 could effectively inhibit SA at a concentration of 0.5 μM or more when not illuminated and at 0.3 μM or more under illumination. cpd.3 showed effective bacteriostasis at 2 μM concentration when not illuminated and 1 μM or more when exposed to light. cpd.4 effectively induced bacteriostasis at 1 μM or more for effective when not illuminated and at 0.3 μM or more during illumination. Therefore, the dual-acting antibacterial effect of the four compounds on SA in the dark followed the order cpd.2 ≥ cpd.1 > cpd.4 > cpd.3. The above results show that cpd.2 has good antibacterial effect on SA with and without illumination, whereas illumination could effectively improve the antibacterial capability of cpd.4.

Figure 1(B) shows the antibacterial effect of cpd.1, 2, 3, and 4 at different concentrations on *E. coli* in the absence of light and with 50 min of illumination. The concentration of cpd.1 needs to be 2 μM in the absence of light to effectively inhibit *E. coli*, and it has the same effect when exposed to light. cpd.2 can effectively inhibit *E. coli* at a concentration of 0.5 μM or more in both illuminated and not illuminated group. cpd.3 effectively induced bacteriostasis at 2 μM when not illuminated and 1 μM or more when exposed to light. Regardless of illumination or dark environment, the concentration of cpd.4 needs to be equal to or greater than 0.5 μM to effectively inhibit bacteria. The dual-acting antibacterial effects of the four compounds on *E. coli* in the absence of light followed the order cpd.2 = cpd.4 > cpd.1 = cpd.3. The above results show that cpd.2 has a good antibacterial effect on *E. coli* with or without illumination.

From the results of SA and *E. coli* bacteriostatic experiments, SA can be inhibited only by using low amounts of cpd.1, cpd.2, and cpd.4, but higher concentrations are required to achieve the same effect in inhibiting *E. coli*. The reason may be the difference in the cell wall structure of Gram-positive and Gram-negative bacteria. The peptidoglycan cell wall of Gram-positive bacteria is porous, whereas the Gram-negative bacteria have a layer of peptidoglycan cell wall between the outer membrane, which also has lipopolysaccharide, and the inner membrane. As a result, most drugs are less likely to penetrate the cell walls of Gram-negative bacteria, resulting in their generally high drug resistance [15,16].

Bacteriostatic experiments revealed that the four studied compounds show dark toxicity to bacteria when not exposed to light and further antibacterial effects when exposed to visible light. The possible cause of dark toxicity is that positively charged compounds can damage the integrity of the cell wall and cell membrane of negatively charged bacteria and ROS generation caused by light, which will cause multiple structural attacks on bacteria. Conventional PDTs require high doses of PS and photocatalysts or UV light to have bacteriostatic effects. The small molecular compounds used in this study have a dual-effect bacteriostatic mechanism, which is conducive to avoiding drug resistance.

In this study, a porous Cs wound dressing with antibacterial effect was further prepared. cpd.2, which has a good inhibitory effect on both *E. coli* and SA, was selected for addition to wound dressings.

N = 3, *p < 0.05, **p < 0.01

### 3.2 Effects of cpd.2 and bFGF on human fibroblast (CG1639) toxicity and cell proliferation

The effects of cpd.2 on the survival rates of CG1639 and of bFGF on the proliferation rate of CG1639 cells were examined by MTT test to investigate whether the porous Cs wound dressing prepared in this study has drug resistance and fibroblast proliferation promotion capability.

**Figure 2.**
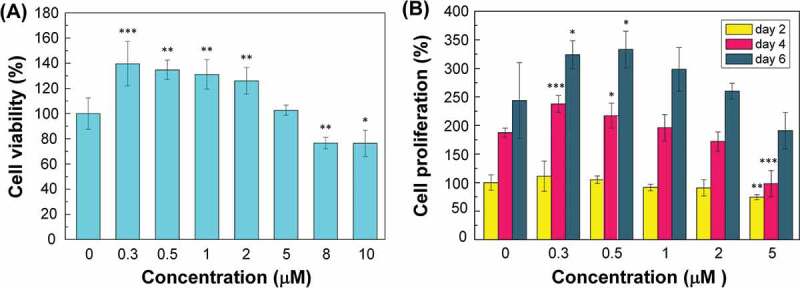
Effect of cpd.2 on the survival rate (A) and proliferation rate (B) of CG1639.

Figure 2(A) shows the effect of cpd.2 at different concentrations on the survival rate of CG1639. The increased dose of cpd.2 to 2 µM maintained a cell survival rate of nearly 125%. When the concentration was 5 µM, the survival rate dropped to 100%, and at 10 µM, the cell survival rate was ca. 80%. Therefore, the proper concentration of cpd.2 which would not affect the cell viability of CG1639 is around 0–5 µM. Furthermore, the low concentration of cpd.2 that has positive effect on the cell viability rate of CG1639 is 0.3–2 µM.

Figure 2(B) shows the effects of different concentrations of cpd.2 on the proliferation rate of CG1639 after 2, 4, and 6 days of culture. Based on the experimental results, cpd.2 could promote cell proliferation at concentrations of 0.3, 0.5, and 1 µM but had no significant effect on cell proliferation at concentration of 2 µM. When the concentration was 5 µM, the concentration of cpd.2 was probably extremely high in the first four days, hence requiring a longer time for the cells to adapt. However, by the sixth day of culture, cell proliferation was similar to that of the control group, indicating that no considerable effect was observed with cell proliferation.

N = 3, *p < 0.05, **p < 0.01, ***p < 0.001.

**Figure 3.**
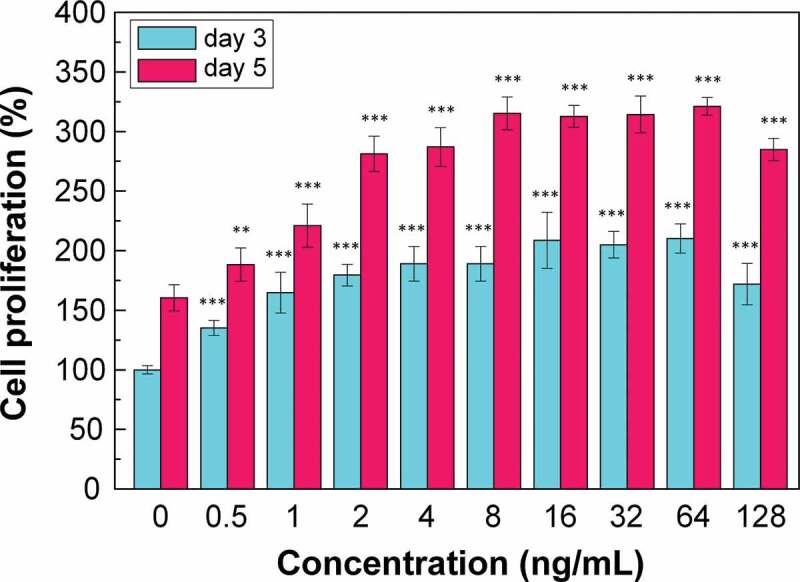
Effect of bFGF on the proliferation rate of CG1639.

Figure 3 shows the effect of different concentrations of bFGF on the proliferation rate of CG1639. Cell proliferation can be effectively promoted when the bFGF concentration was 0.5–128 ng/mL. When the bFGF concentration was between 0.5–2 ng/mL, the promotion effect increased with the bFGF concentration. When the bFGF concentration was 2–64 ng/mL, the promotion effect on cell proliferation was saturated. When the bFGF concentration was 128 ng/mL, the promotion effect on cell proliferation decreased.

N = 3, *p < 0.05, **p < 0.01, ***p < 0.001.

Increasing the concentration of cpd.2 can effectively improve its antibacterial effect. Therefore, in subsequent experiments, 5 µM, which causes no effect on the survival of CG1639 and has a better antibacterial effect, was selected as the dressing concentration. bFGF was added at a concentration of 2 ng/mL.

**Figure 4.**
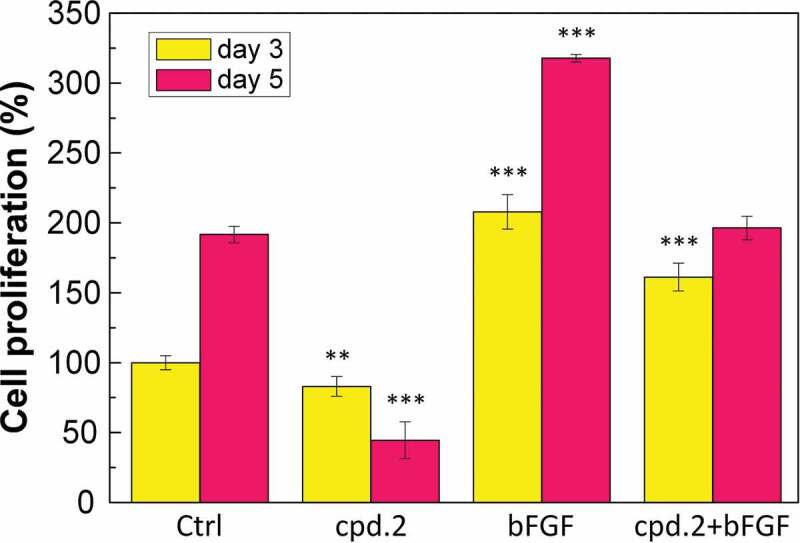
Effects of cpd.2 and bFGF on the proliferation rate of CG1639.

Figure 4 shows the effect of cpd.2 at a concentration of 5 µM and bFGF at a concentration of 2 ng/mL on the proliferation rate of CG1639. After three days of cell culture, cpd.2 reduced the cell survival rate to 83.0%, whereas bFGF increased the cell survival rate to 207.8%. cpd.2 + bFGF improved the cell survival rate to 161.2%. The experimental results show that cpd.2 can reduce the survival rate of CG1639, but the addition of bFGF can significantly promote the proliferation of CG1639.

N = 3, *p < 0.05, **p < 0.01, ***p < 0.001.

### 3.3 Characterization of the porous Cs dressing

The use of organic solvents or excessive cross-linking in the preparation of antibacterial dressings must prevent cpd.2 from contacting pathogens to avoid affecting the antibacterial effect of cpd.2. At the same time, care must be taken to maintain the acid–base characteristics of the material. A common method for preparing a porous Cs material is dissolving the Cs particles with acetic acid, adding a cross-linking agent, freeze drying, and finally soaking in a sodium hydroxide solution for alkaline washing and then drying. However, regardless of the step cpd.2 where is added in this process, cpd.2 degradation may still occur. Therefore, in our fabrication process, sodium hydroxide was first dropped into the Cs acetic acid solution until the pH was 7, and 5 µM cpd.2 was added. Finally, freeze drying was performed to form a porous Cs dressing. The main reason for the formation of a porous Cs structure is that Cs will form ammonium ions (-NH^3+^) in acetic acid and chemically react with sodium hydroxide. The -OH of sodium hydroxide will grab H^+^ from the free -NH^3+^ and reduce Cs to -NH_2_. Then, the Cs molecules will generate hydrogen bonds to form a gel with a network structure.

**Figure 5.**
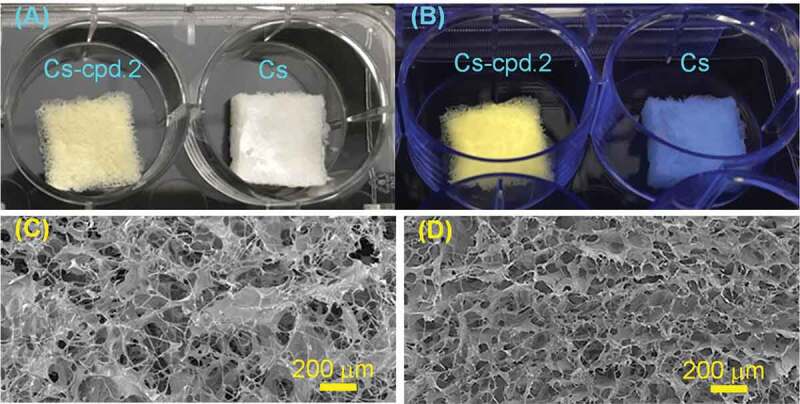
(A) Photographs of the Cs-cpd.2 and Cs dressings; (B) Cs-cpd.2 dressing emitting fluorescence under UV irradiation; (C) SEM image of the Cs-cpd.2 dressing; (D) SEM image of the pure Cs dressing.

Figure 5(A) shows the Cs-cpd.2 and pure Cs dressings. The Cs-cpd.2 dressing can emit fluorescence under ultraviolet light irradiation (Figure 5(B)), which indicates that the fluorescent properties of cpd.2 was not degraded in this dressing process. From the SEM images in Figure 5(C) and 5(D), the interconnected pore structures in the dressing can be observed. As shown in Table 1, the average pore size of Cs is 100 ± 40 µm, the porosity is 90% ± 9%, and the water absorption is 923% ± 15%. The average pore size of Cs-cpd.2 is 90 ± 30 µm, the porosity is 87% ± 14%, and the water absorption rate is 996% ± 102%. The best wound dressing pore size is between 20 and 120 µm, because such a pore size allows cell migration and effective cell adhesion [17]. High porosity and high water absorption enable the absorption of a large amount of wound exudate and help nutrient and oxygen transfer of cells attached to the dressing [18]. The experimental results show that our dressing method can obtain wound dressings with appropriate pore size, high porosity, and high-water absorption.

**Table 1.** Cs and Cs-cpd.2 porous property analysis.

	Average pore size (µm)	Porosity (%)	Water absorption rate (%)
Cs	100 ± 40	90 ± 9	923 ± 15
Cs-cpd2	90 ± 30	87 ± 14	996 ± 102

### 3.4 Antibacterial effect of the porous Cs dressing

According to JIS-L-1902, L ≥ 0 means that the material can kill skin infection and harmful bacteria on the material; S ≥ 2 means that the material has bacterial growth-inhibiting and deodorizing effects. SA is the most commonly isolated strain in infectious wounds. Thus, the antibacterial assay of SA can determine whether Cs-cpd.2 is useful in preventing and treating wound infection. The experimental results in Table 2 show that the Cs dressing was covered with SA when the Cs and Cs-cpd.2 dressings were inoculated with SA at a concentration of 1.45 × 10^5^ CFU/mL and cultured for 24 h. However, no evident colony formation was observed on the Cs-cpd.2 dressing (Figure S1). After calculation, the proliferation values (F) of Cs dressing without and with light were 6.41 and 6.99, indicating that SA on Cs had grown by 10^6^ folds after 24 h. Thus, no bacteriostatic effect was observed. Cs-cpd.2 has a good antibacterial effect with or without light. The experimental results indicate that the Cs-cpd.2 dressing has antibacterial effects on the wound.

**Table 2.** Antibacterial effect of Cs and Cs-cpd2 dressings on SA.

**Inoculum concentration**	1.45 × 10^5^ (CFU/mL)	
CFU on Cs dressing (0 h)	5.5 × 10^4^	
CFU of Cs-cpd.2 dressing (0 h)	4.2 × 10^4^	
	**Dark**	**Light**
**CFU on Cs dressing (24 h)**	1.40 × 10^11^	5.44 × 10^11^
*Growth value* (F)	6.41	6.99
**CFU on Cs-cpd.2 dressing (24 h)**	7.68 × 10^5^	1.67 × 10^4^
*Bactericidal activity* (L)	−1.15	0.52
*Antibacterial activity value* (S)	5.15	7.40

### 3.5 Toxicity of porous Cs dressing to CG1639

**Figure 6.**
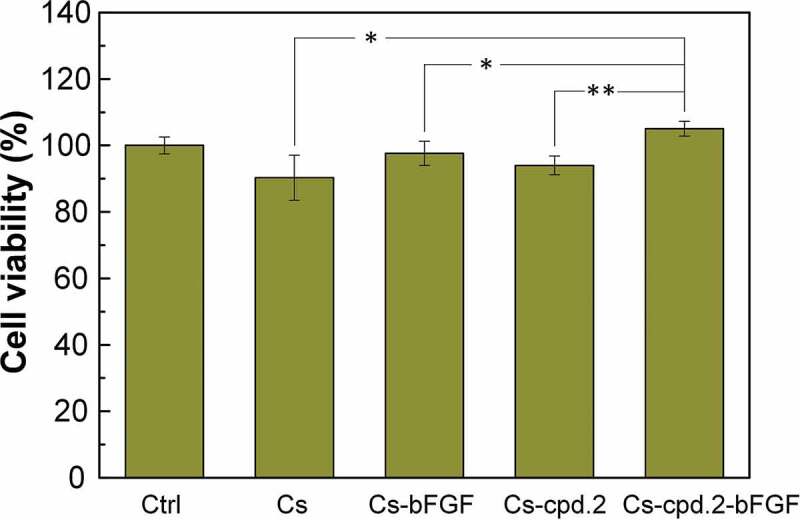
Cytotoxicity of Cs, Cs-cpd.2, and bFGF to CG1639.

The materials were placed in CG1639-coated 24 wells, and their cell viability was measured after 24 h to examine the cytotoxicity of Cs, Cs-cpd.2, and bFGF to CG1639. The experimental results shown in Figure 6 indicate that the cell survival rates of the Cs, Cs-bFGF, Cs-cpd.2, and Cs-cpd.2-bFGF dressings were 90%, 98%, 94%, and 105%, respectively. Compared with other experimental groups, pure Cs dressing reduced the cell survival rate, but the addition of cpd.2 and bFGF resulted in an increase.

N = 3, *p < 0.05, **p < 0.01, ***p < 0.001.

### 3.6 Effect of porous Cs dressing on CG1639 proliferation

**Figure 7.**
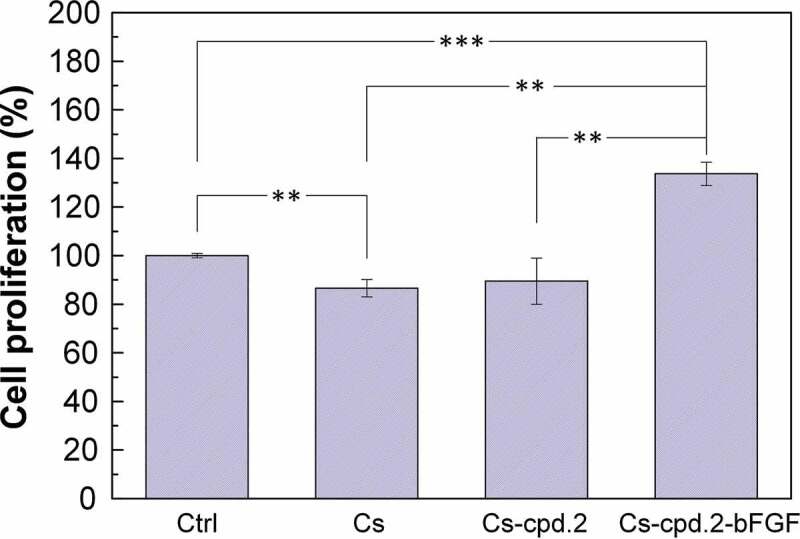
Effects of Cs, Cs-cpd2, and bFGF on CG1639 proliferation.

The materials were placed in CG1639-covered 24 wells, and the cell survival rate was measured after three days of culture to examine the effects of Cs, Cs-cpd2, and bFGF on the proliferation of CG1639. Figure 7 reveals that the cell proliferation rates of the Cs, Cs-cpd2, and Cs-cpd.2-bFGF dressings were 87%, 90%, and 134%, respectively. The experimental results revealed that pure Cs dressing alone was not conducive to CG1639 proliferation, and no significant improvement was observed after adding cpd.2. However, cell proliferation can be significantly promoted when cpd.2 and bFGF are added together.

N = 3, *p < 0.05, **p < 0.01, ***p < 0.001.

## 4. Conclusions

In this study, a dual-acting antibacterial porous Cs film embedded with a photosensitive small molecule (cpd.2) was fabricated using the freeze-drying method. The average pore sizes of pure Cs and Cs-cpd.2 dressings were measured to 100 ± 40 and 90 ± 30 µm, respectively. Both dressings have high porosity and moisture absorption effect, hence enhancing the absorption of wound exudate. The dual-acting antibacterial property of the fabricated Cs-cpd.2 dressing was demonstrated by its good bactericidal and bacteriostatic effects on SA under light condition and antibacterial effect when not illuminated. When the concentration of cpd.2 was 0.3, 0.5 and 1 μM, the viability rate and proliferation rate of CG1639 were higher than those of the control group, indicating that 0.3–1 μM concentrations of cpd.2 can improve cell attachment and proliferation. Furthermore, Cs-cpd.2 that contained small molecular compound with a concentration of 0.3–1 μM can improve the cell viability rate and cell proliferation rate of human fibroblast CG1639. Cs-cpd.2 can significantly promote cell proliferation when the small molecular compound and the bFGF are added together. The dual-acting antibacterial property of the proposed Cs-cpd.2 dressing makes it highly feasible for photodynamic therapy (PDT) and clinical wound dressing applications.

## Supplementary Material

Supplemental Material
