# Supplementary material for: Dual-acting antibacterial porous chitosan film embedded with a photosensitizer
Source: Sci Technol Adv Mater. 2020 Aug 3;21(1):562–72. doi: 10.1080/14686996.2020.1795431 (PMC7476534; doi:10.1080/14686996.2020.1795431)
Supplement: Supplemental Material [file TSTA_A_1795431_SM4711.pdf]

Supporting Information

Dual-acting antibacterial film of porous chitosan embedded with small molecular compound

1. Small molecule compounds used as used as the photodynamic agents

Compound 1 (cpd.1)

1,6-bis((E)-2-(N-methylpyridinium-4-yl)vinyl)-2-(12-(N-methylpiperidinium-1-yl)dodecyloxy)naphthalen triiodide as reported in the previous literature .[23]

Compound 2 (cpd.2)

3,6-bis(1-methyl-4-vinylpyridinium iodide)-9-(1-(1-methyl-piperidinium iodide) dodecyl) carbazole as reported in the previous literature.[24]

Compound 3 (cpd.3)

3,6 Bis --(1 methyl 2 vinylpyridium iodide) 9 --(1 --(1 methyl Piperidinium iodide)dodecyl) carbazole as reported in the previous literature.[25]

Compound 4 (cpd.4)

3,6-bis(1-methyl-4-vinylpyridinium iodide)-10-(1-(1-methyl-piperidinium iodide) dodecyl) phenothiazine

Compound 4 was synthesized from *3,7-Dibromo-10H-phenothiazine* (2 mmol) through 10 position substituting by sodium hydride (3.5 mmol 0.295 g) in DMF (20 mL) under nitrogen gas condition. 1,12-dibromododecane (100 mM) were then added and the mixtures were refluxed for 12 h . After cooling and quenching the excess sodium hydride with methanol, the solution was extracted with H_2O/ethyl acetate twice and the organic layer dried by $MgSO_4$. The products (*3,7-Dibromo-10-(1-bromo dodecyl) phenothiazine*) were collected by flash column (silica, hexane/ethyl acetate. 2/1, v/v). Next step was conveniently obtained by way of refluxing this intermedium (2 mmol) and piperidine (0.5 mL) in ethanol (20 mL) for 6 h with trace of sodium iodide. The solvent was evaporated in vacuum and the residue purified via column chromatography (silica, hexane/ethyl acetate. 1/2, v/v) to collect the yellow products (*3,7-Dibromo-10-(1-(piperidin-1-yl) dodecyl) phenothiazine* which then coupled with 4-vinylpyridine at mixed powders of Palladium (II) acetate/tri-*o*-tolylphosphine under the triethylamine/ acetonitrile solvent pairs in high pressure system. The bottle was sealed after bubbling 10 min with nitrogen. After keeping the system under ~105°C for two days, the precipitant was collected and then extracted with H_2O/CH_2Cl_2 twice. The organic layer was dried by $MgSO_4$ and then filtered. Crude products were purified by flash column chromatography with acetone/n hexane as eluent gradient to collect the

compound *3,7 Bis-(4-vinylpyridine) -10-(1-(piperidin-1-yl)dodecyl) phenothiazine*. Finally, the orange red powders (compound 4) were collected in very good yield after refluxing compound *3,7 Bis-(4-vinylpyridine) -10-(1-(piperidin-1-yl)dodecyl) phenothiazine* with excess CH_3I in DMF. Data: ^{1}H NMR (DMSO-d6): δ= 9.46 (s, 1H), 8.76 (d, J=6Hz, 4H), 8.06 (d , J=6Hz, 4H), 7.79 (d, J=16Hz, 2H), 7.34 (m, 4H), 7.28 (d, J=16Hz, 2H), 6.71 (d, J=6Hz, 4H), 4.52 (t, J = 6.8 Hz, 2H), 4.44 (s, 6H), 3.26 (m, 6H), 2.95 (s, 3H), 1.81 (m, 2H), 1.75 (m, 4H), 1.61 (m, 2H), 1.51 (m, 2H), 1.28 (m, 4H), 1.20 (m, 12H). m/e mass: $[M+H]^+$: 1155.22, Found: 1155.78. EA: Anal. Calcd. For $C_{46}H_{61}I_3N_4S$: C, 51.03; H, 5.68; I, 35.16; N, 5.17; S, 2.96. compound 4 ·$4H_2O$: $C_{46}H_{69}I_3N_4O_4S$: C, 47.84; H, 6.02; I, 32.97; N, 4.85; O, 5.54; S, 2.78 Found: C, 47.90; H, 6.08; N, 4.85.

2. S. aureus cultured on Cs dressing and Cs-cpd2 dressing

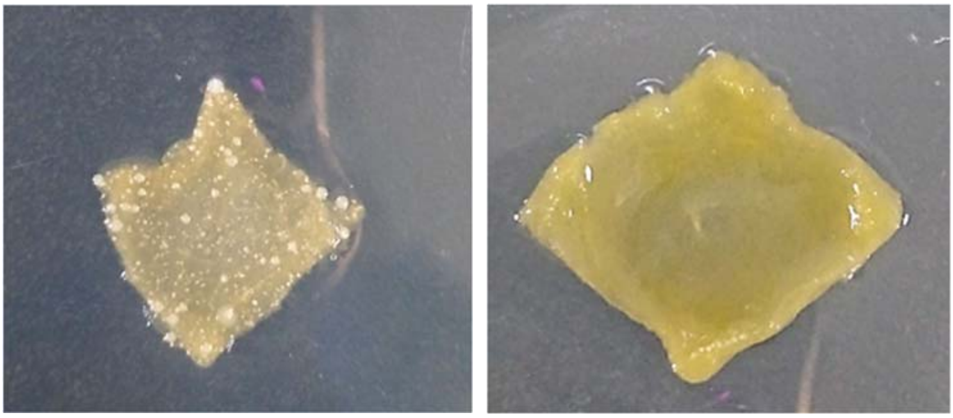

(A) (B)

Fig. S1. S. aureus cultured on Cs dressing and Cs-cpd2 dressing. (A) Cs dressing inoculated S. aureus for 24 h; (B) Cs- cpd.2 dressing inoculated S. aureus for 24 h
